# Hagfish slime and mucin flow properties and their implications for defense

**DOI:** 10.1038/srep30371

**Published:** 2016-07-27

**Authors:** Lukas Böni, Peter Fischer, Lukas Böcker, Simon Kuster, Patrick A. Rühs

**Affiliations:** 1 Department of Health Science and Technology, ETH Zürich, 8092 Zürich, Switzerland; 2 Department of Materials, ETH Zürich, 8093 Zürich, Switzerland

## Abstract

When hagfish (*Myxinidae*) are attacked by predators, they form a dilute, elastic, and cohesive defensive slime made of mucins and protein threads. In this study we propose a link between flow behavior and defense mechanism of hagfish slime. Oscillatory rheological measurements reveal that hagfish slime forms viscoelastic networks at low concentrations. Mucins alone did not contribute viscoelasticity, however in shear flow, viscosity was observed. The unidirectional flow, experienced by hagfish slime during suction feeding by predators, was mimicked with extensional rheology. Elongational stresses were found to increase mucin viscosity. The resulting higher resistance to flow could support clogging of the attacker’s gills. Shear flow in contrast decreases the slime viscosity by mucin aggregation and leads to a collapse of the slime network. Hagfish may benefit from this collapse when trapped in their own slime and facing suffocation by tying a sliding knot with their body to shear off the slime. This removal could be facilitated by the apparent shear thinning behavior of the slime. Therefore hagfish slime, thickening in elongation and thinning in shear, presents a sophisticated natural high water content gel with flow properties that may be beneficial for both, defense and escape.

Various animals produce slime in locomotion and adhesion^1^. By contrast, the hagfish forms a mucus-based slime with long protein threads as a very effective defense mechanism against predators^2^. In contact with seawater, the slime generates from tiny quantities of a glandular exudate, released from ventrolateral pores (Fig. 1a,b). The exudate (Fig. 1d) is composed of three components: vesicles containing mucin (Fig. 1c), protein threads coiled up in skeins (Fig. 1e), and a residual fluid^3^. Upon contact with water, a cascade of physico-chemical events is triggered in the exudate and within milliseconds vast amounts of slime are produced. The vesicles rupture through the influx of water^4^ and the protein skeins unravel and expand to long threads^5^,^6^. The mucins form strands under the influence of convective mixing and attach to the unravelling threads^7^.

The threads impart outstanding physical properties and make the slime an elastic and coherent soft gel with a complex network structure, consisting of ultra-long protein threads (≈15 cm) and a hydrated mucus part^2^,^8^,^9^. The threads are composed of intermediate filament proteins^10^ that undergo a so called *α*-helix to *β*-sheet transition when subjected to large deformation, leading to substantially improved mechanical properties similar to spider silk^11^. The slime is very dilute, containing 99.996% water which is physically confined between the threads and the mucins and thus only transiently retained^3^. Hagfish are preyed upon by a variety of aquatic animals, exposing the fish and the formed slime to feeding mechanisms such as biting or suction (Fig. 2a). Suction feeders rapidly expand their buccal cavity, thereby creating a unidirectional flow of water that engages the prey and draws it into the mouth^12^,^13^. As shown by Zintzen *et al.*^14^, in all observed cases of predation, the hagfish were able to free themselves from the attacker by gill-clogging^2^, suggesting that their defense mechanism is very efficient and crucial for their survival. Being such an outstanding defense, the slime has a major drawback for the hagfish. If they were not able to free themselves from their own slime, they might self-asphyxiate. Therefore, hagfish can tie a sliding knot with their own body to strip off the slime and thus avoid self-entanglement (Fig. 2b)^15^,^16^. Escaping slime by knotting seems important as hagfish do not only secrete slime when attacked, but also when feeding or injured^16^.

Despite the remarkable properties of hagfish slime, the flow behavior behind its defensive properties and the individual contributions of each slime component are largely unknown. The rheology of slime was studied previously^9^,^17^,^18^, however the flow properties of the slime were not linked to its main purpose, i.e. its protecting capacity. Therefore, we studied the rheological properties of hagfish slime and hagfish mucin in elongational, shear, and oscillatory flow to investigate the role of hagfish slime in defense and escape. The slime has a unique network structure, as small concentrations (0.01 wt%) are sufficient to develop resistance features against flow such as viscosity and elasticity. Using a rheological approach, we propose that the flow properties of the slime seem beneficial to its ability to clog the gills of predators (thickening in elongation), and its ability to be shed by hagfish that get trapped within the slime (thinning in shear).

## Results and Discussion

The flow behavior of hagfish slime was measured with rheology to study its implications for the defense mechanism. In the first part the influence of the threads on the slime network properties were studied. In the second and third part the effect of shear and elongational forces on the slime were investigated. Based on our measurements, the apparent shear thinning and elongational thickening flow properties were linked to biological implications for hagfish defense and escape strategies.

### Hagfish slime viscoelasticity

**Figure 3:**
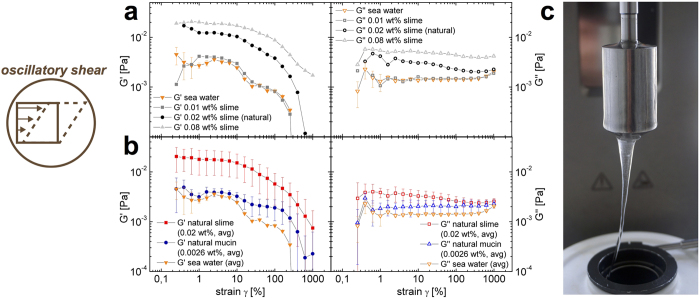
Oscillatory shear rheology of hagfish slime and mucin. (**a**) Amplitude sweep of hagfish slime (G′ left and G″ right) at varying concentrations (0.01 to 0.08 wt%, 0.01 wt% being the natural slime concentration) at a fixed angular frequency of *ω* = 1 rad/s. (**b**) Amplitude sweep (G′ left and G″ right) of five averaged measurements of hagfish slime (0.02 wt% being the natural concentration), hagfish mucin (0.0026 wt% being the natural mucin concentration), and seawater as a reference at a fixed angular frequency of *ω* = 1 rad/s. (**c**) In oscillation and simple shear the rheological properties of hagfish slime were measured with a Couette geometry.

One of the most striking features of hagfish slime is the low concentration needed to gel vast quantities of water^3^. We therefore investigated the rationale behind the natural economic use of material by gradually increasing the slime concentration and studied the contribution of threads and mucins to the network properties. With oscillatory rheology, the network properties of hagfish slime and mucin were measured at exudate concentrations from 0.01 to 0.08 wt% and compared to naturally occurring concentrations of mucin in 0.01 wt% slime. A concentration of 0.01 to 0.02 wt%, being the natural concentration^3^, is sufficient to develop elastic features with a higher storage modulus G′ than loss modulus G″ (Fig. 3a, see Supplementary Video 2). This is unique, as most biological hydrogels require a much higher concentration to exhibit gel-like features^19^. A concentration of 0.01 wt% exudate was found to be the lowest functional concentration as it visibly still gelled the entire system but showed a viscoelastic signal close to water, which also exhibits an apparent elastic modulus due to surface tension effects (Fig. 3b, left). Ewoldt *et al.*^20^ reported the considerable rheological challenges (sample and instrument inertia, instrument resolution, boundary effects) in determining the underlying material functions of soft and water-based bio-materials, such as hagfish slime. However, although in this study a more than fourfold lower concentration was used than by Ewoldt *et al.*^9^ (0.83 mg/ml by Ewoldt *et al.*, corresponding to 0.083 wt%), an almost identical softness for hagfish slime (G′ ≈ 0.02 Pa) was measured. When the concentration was increased to 0.08 wt%, the linear viscoelastic modulus G′ still remained largely constant. This finding suggests that hagfish slime is an inherently soft material, regardless the concentration.

In 0.01 wt% slime, about 20% is mucin^3^, therefore we evaluated to what extent the mucins contribute to the rheological response. We found that the apparent elasticity of the mucin fraction (20% of 0.01 wt%) is nearly identical to seawater. This can be seen in the substantially higher moduli of the slime compared to mucin in Fig. 3b showing the average of five measurements for slime, mucin, and water, respectively. At a concentration of 0.0026 wt% the mucins do not seem to form a network across the entire slime or the used measurement technique is not sensitive enough. This data supports the theory of Fudge *et al.*^3^, who suggested that hagfish mucins do not form a cross-linked network throughout the slime but are rather heterogeneously distributed in discrete networks. Therefore, to have a viscoelastic network, the threads seem to be important for the overall viscoelasticity.

### Shear thinning of hagfish slime

**Figure 4:**
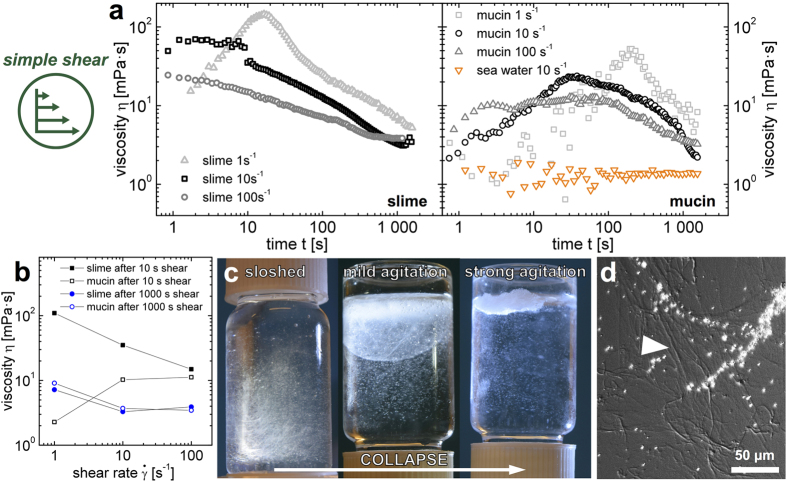
Shear induced collapse of hagfish slime and aggregation of mucin. (**a**) Simple shear of hagfish slime (left) and hagfish mucin (right) mimicking the flow properties during knot sliding at concentrations of 0.02 wt% for slime and 0.0026 wt% for mucin, respectively. Hagfish slime viscosity was measured at constant shear rates (1, 10, 100 s^−1^) over a period of 30 minutes. (**b**) The viscosity values plotted as a function of shear rate, showing the apparent shear thinning behavior of hagfish slime and the influence of time on the viscosity. (**c**) Agitation causing a collapse of the slime network by thread association. (**d**) Differential interference contrast (DIC) micrograph of a mucin floc after shear of a mucin solution, showing mucin aggregated into thread-like structures (arrowhead).

Hagfish have to escape their own slime to avoid suffocation. They can form a sliding knot with their body to shear off the slime. The influence of simple shear flow on the slime properties is measured at shear rates of 1, 10, and 100 *s*^−1^ corresponding to the range of natural shear rates (see Supplementary Note, a). As hagfish slime is highly sensitive to its mechanical history^3^,^8^, no pre-shear experiments were performed. Hagfish slime shows an apparent shear thinning behavior (Fig. 4a, left and Fig. 4b) but in contrast to some other shear thinning solutions, with shear thinning being an intrinsic property of a homogeneous material, the slime viscosity decreases even at constant shear rates. This effect can be attributed to the coiling of threads around the geometry^9^ and thread association, leading to a collapse of the slime network (Fig. 4c) and consequently to a phase separation of a condensed gel network separating from watery remains. A collapse of the network due to shear is also visible in Fig. 4a, right where the mucin fraction was measured without threads. We propose that the mucin fraction is not stable against shear, forming aggregates (Fig. 4d), which lowers the viscosity (see Supplementary Video 3). The tendency of mucins to aggregate and thus cause a gel-sol transition is known and mainly attributed to inter-molecular hydrophobic interactions among protein segments^21^,^22^. We propose that given their large size and their high protein content^23^, shear flow causes hagfish mucin to aggregate by facilitating inter-molecular hydrophobic interactions. The aggregating mucin fraction supports a collapse of the slime network by thread association. This collapse can be circumvented, as was shown in a previous study^17^, with a network stabilized by negatively charged biopolymers.

### Elongational thickening during suction feeding

**Figure 5:**
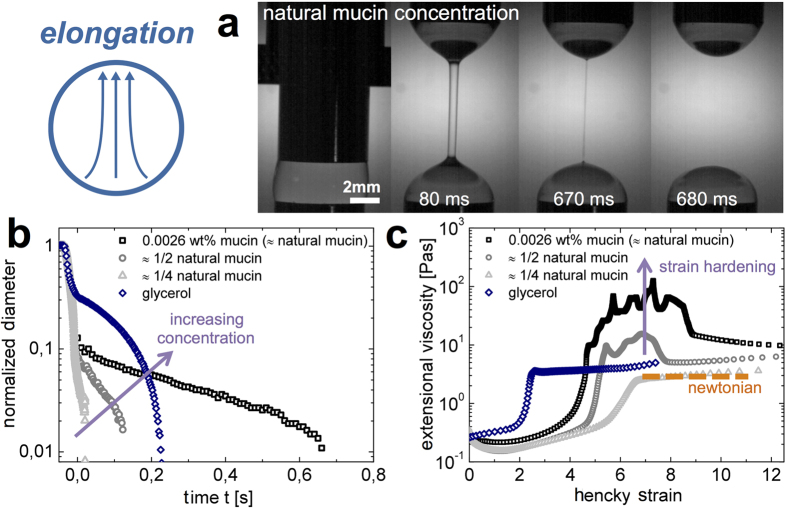
Capillary breakup extensional rheology (CaBER) measurements mimicking the extensional flow experienced by hagfish slime during suction predation. (**a**) A liquid filament thinning event of hagfish mucin (natural concentration) at t: 0, 80, 670, 680 ms. (**b**) The normalized filament diameter of hagfish mucin at a natural concentration, 50%, and 25% of the natural concentration as a function of time. Glycerol is provided as a Newtonian standard. (**c**) Extensional viscosity of hagfish mucin as a function of Hencky strain based on the data provided in (**b**). The orange dashed line indicates a Newtonian profile as observed for glycerol. An exemplary filament thinning event of natural hagfish mucin is provided in the Supplementary Video 5.

When hagfish are predated through suction feeding (for suction feeding predators on hagfish^14^,^24^ see Supplementary Note, b), the slime is stretched by elongational, unidirectional flow^25^,^26^. In addition, extensional flow is also likely to be important for the development and formation of the slime^7^. Extensional measurements with hagfish slime threads revealed that threads are very elastic in extension^27^,^28^,^29^. Additionally, distinct elastic features of hagfish mucins can be observed in hagfish slime (see Supplementary Video 4). To address the effect of the mucin fraction, we measured the extensional rheology at the natural concentration of hagfish mucin and at lower concentrations to determine their role in the flow properties of hagfish slime (Fig. 5a) using Capillary breakup extensional rheology (CaBER). A strike time of 50 ms corresponding to a natural prey-sucking time was chosen^25^,^26^,^30^.

In Fig. 5b the liquid filament thinning events of three mucin concentrations in seawater are shown. At the natural mucin concentration, a delayed breakup of the liquid filament can be observed. By contrast, a lower mucin concentration (25% of the natural mucin concentration) coincides well with a Newtonian profile, suggesting that this concentration is very dilute and the mucin thread thinning is mainly ruled by capillary forces and given the low concentration possibly also by surface tension forces. A loss of elastic stresses and a Newtonian behavior with a decreasing mucin concentration is also observed in the corresponding extensional viscosity curves in Fig. 5c. A pronounced strain hardening or an increase in extensional viscosity with strain is observed, which is attributed to a strong resistance of flexible polymer molecules to extensional flow^31^. Strain hardening is a known phenomenon for mucus systems^32^,^33^,^34^,^35^ and is thought to arise from peculiarities of the extension kinematics, and to be related to a deferred disentanglement process^36^. A similar strain stiffening of hagfish slime was described by Ewoldt *et al.*^9^ from oscillatory shear measurements, who likewise suggested that mucins, as non-linear elastic network components, are strain-stiffening. As shown in Fig. 5b,c, a strain hardening can be observed when the concentration of mucin is increased as strain hardening depends on the molecular weight and concentration of polymer in solution^31^. In the case of hagfish slime (with threads), mucin is entrained in defined regions due to the threads. We suggest that in combination with the elastic threads, the viscosity is even further increased, causing an elongational thickening during extensional stresses. The resulting resistance against flow has a strong influence on the elastic behavior of hagfish slime as presented in Fig. 3. An increased viscosity thus might reduce the water flow at the predators’ gills, supporting gill clogging.

## Conclusion and Biological Implications

Hagfish are able to form viscoelastic slime networks at very low concentrations (0.01 wt%) with distinct rheological properties in oscillation, simple shear, and extension. The low concentration allows for an economic use of exudate but results in a short-lived and soft gel. Nevertheless, the short-longevity of the slime may be of advantage for the hagfish as it can escape from its own slime. Furthermore, the slime forms within seconds, allowing a fast response upon attack without large energetic triggers for slime development, which is in contrast to most hydrogels that require substantial energy input for network formation. We suggest, that the balance between gel structure and time/energy needed to form this structure is favorable for the defense situation. Using a rheological approach, we propose that not only the viscosity but also the elasticity of hagfish slime is largely determined by the mucin fraction and its synergistic interplay with the threads. So far only the viscous behavior of hagfish mucins was reported^3^. Although we could not detect mucin elasticity using oscillatory rheology, we base our assumption on their distinct extensional elastic properties. Hagfish mucins, being large biopolymers^23^, are known to attach to the threads^7^. By this anchoring additional network points are created, thus decreasing the overlap concentration of the mucin dispersion. We propose that the threads provide long range properties such as extensibility and cohesiveness of the slime, prevent mucin wash-out^2^,^3^,^18^, and allow the mucin to exhibit viscoelasticity by supplying anchoring points. Thus, when combined with the threads, the mucin fraction can establish viscoelastic properties, despite the low natural concentration.

**Figure 6:**
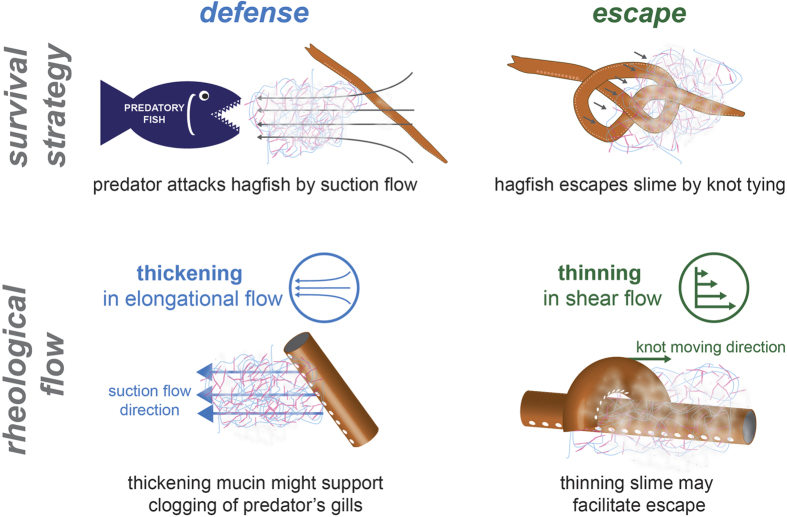
Hagfish survival strategies and their underlying flow principles. During predation, hagfish secrete and form large quantities of slime as a highly effective defense mechanism. Many predators hunt by suction feeding, where a strong unidirectional elongational flow draws in the prey. When the slime is subjected to elongational flow, the viscosity of the hagfish mucin increases, which might support clogging of the predator’s gills. Facing suffocation and entrapment in their own slime, hagfish shear off the slime by moving a knot across their body. The applied shear leads to a viscosity decrease and a collapse of the slime, which may facilitate the escape and prevent self-asphyxiation.

The slime flow behavior in shear and extension seem beneficial for the biological survival strategies of hagfish slime (see Fig. 6). Predators attacking hagfish often use suction feeding^14^. We propose that suction flow has two effects on the slime. First, flow supports the slime formation^7^. Second, extensional flow, created through suction feeding induces an increase in extensional viscosity of hagfish mucin. An increased viscosity reduces the water flow and thus could support gill clogging. Furthermore, the cohesiveness provided by the threads could also be important for predator gill clogging or to defend against biting predators^2^. To avoid self-asphyxiation, hagfish are able to form a knot with their body to release themselves from their slime^15^. The shear thinning behavior of the slime may be helpful in this situation. Yet, other flow phenomena such as lubrication and slip might also be important. Shear forces eventually lead to a collapse of the slime network, which is supported by mucin aggregation.

However, some questions and limitations of this study remain to be addressed in order to draw further conclusions on the functions in life and the selective pressures that have led to the slime’s physical properties. Knowing the relative amounts of extension to shear during suction feeding events will provide deeper insights in the opposite behavior of the slime under the respective flow conditions. Additionally, the timing of slime formation and possible changes of the slime during formation might be critical as the slime could enter the mouth of a predator in an incompletely deployed state. Therefore, further studies will extend the basis for hypotheses on the evolution of hagfish slime as a defense mechanism. In summary, we propose that hagfish slime flow properties, thickening in elongation and thinning in shear, may be beneficial for both, escape and defense. Besides the biological significance, the synergistic effect between macroscopic extensible threads and microscopic extensible mucin molecules might allow the formulation of novel, bio-inspired, and functionalized hydrogels with an enormous water holding capacity.

## Methods

### Hagfish exudate sampling and stabilization

Atlantic hagfish (*M. glutinosa*) were fished and provided by the staff of the Atlanterhavsparken in Ålesund, Norway. Sampling was carried out in accordance to the approved ethical application by the Forsøksdyrutvalget (FOTS ID 6912) and followed the protocol of Herr *et al.*^6^. In brief, the fish were placed in a 10 l bucket of fresh and cold seawater and anaesthetized using a 1:9 mixture of clove bud oil (Sigma, Switzerland) to ethanol at a concentration of 1 ml/l. Once unresponsive to touch, the hagfish were placed on a dissection tray, blotted dry and electrically stimulated (80 Hz, 18 V, HPG1, Velleman Instruments) on the venter, which causes the muscles to contract and the exudate to be expelled from the pores. The released exudate was collected and stabilized under MCT oil (medium chain triglycerides, Delios GmbH, Germany) for whole slime measurements or in a high osmolarity buffer (0.9 M sodium citrate and 0.1 M PIPES at pH 6.7, 0.02% sodium azide and protease inhibitor)^6^ for mucin measurements and stored at 4 °C. Sampling was carried out under the supervision of Møreforsking. After sampling, the fish were transferred to a recovery bath. Import of the samples was granted by the Swiss Federal Food Safety and Veterinary Office (FSVO) and export was granted by Norwegian Seafood Council. To form a slime mass for rheology measurements, a specific volume of MCT stabilized exudate was placed on the bottom of a 20 ml glass flask with a micropipette. Seawater (Norway) that was sterile filtered (0.2 *μ*m PA-20/25 filter, MN, Germany) was added and the samples were mixed by gentle sloshing heads over eight times, as similarly performed by Ewoldt *et al.*^9^. The resulting exudate concentration of the measurements was determined according to the assumption of Ewoldt *et al.*^9^ (density of the exudate is close to 1 g/ml, as about 66% of the exudate mass is water^3^). Measurements with hagfish mucins were done by mixing mucin vesicles solution with seawater. The vesicle solution was prepared according to the protocol of Salo *et al.*^23^. In brief, exudate stabilized in high osmolarity buffer was filtered through a series (60, 41, and 20 *μ*m) of nylon mesh filters (Merck) to separate the small mucin vesicles from the skeins. To obtain a high vesicle concentration, the filtrate was centrifuged at 2000 g for 10 min and the supernatant discarded. The mucin content of the vesicle solution was determined in triplicates by dialysis (25 kDa MWCO, SpectraPor, USA), dialyzing 0.5 ml of the vesicle solution against three batches of milliQ water (12 h each) and subsequent freeze drying to determine the dry weight. The mucin content of the stock solution was 2.6 ± 0.8 mg/ml. All mucin measurements were performed at a constant citrate/PIPES concentration of 10 mM in seawater to ensure a constant ionic strength.

### Shear and oscillatory rheology

A shear rheometer (Physica MCR501, MCR702, Anton Paar, Austria) with a Couette geometry (CC27, Anton Paar, Austria) was used for shear experiments. Amplitude sweeps were performed at a fixed frequency *ω* = 1 rad/s. Shear viscosity was measured by applying constant shear rates of 1, 10, and 100 s^−1^ over a period of 30 min. A shear rate dependent viscosity was obtained by plotting the viscosity of the constant shear rate experiments over the applied shear rate at a given time. Measurements were performed at 10 °C.

### Extensional rheology

Capillary breakup extensional rheometry (CaBER) measurements were carried out on a HAAKE CaBER 1 (Thermo Haake, Thermo Fisher Scientific). For CaBER experiments only the hagfish mucin was used due to the large size of the protein threads. The sample was loaded between the two plates, which were separated with an initial step strain (50 ms). A liquid bridge was formed between the two cylindrical test fixtures followed by a self-driven uniaxial extensional flow, which leads to a breakup of the filament. The response of a fluid following an axial step-strain is encoded in an apparent transient elongational viscosity function 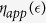, which can be determined by measuring the change of the filament diameter *D*_*mid*_ (or filament radius *R*_*mid*_) and strain rate 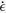 as a function of time:


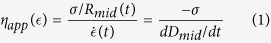


where *σ* is the surface tension. The resulting system Hencky strain 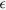 is defined as 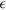 = 2 ln(*D*_0_/*D*_*mid*_(*t*)) with *D*_0_ being the initial diameter of the fluid thread before stretching^37^,^38^,^39^. The measurements were performed at an initial gap of 3 mm and a sample final height of 12.03 mm with an initial aspect ratio of 1 and a final aspect ratio of 4.01. The measurements were conducted at room temperature.

### Microscopy

Light microscopy images were captured on a Nikon Diaphot (Nikon, Japan) and analyzed with the NIS elements D3.0 software. DIC (Differential interference contrast) microscopy was performed on a Leica DM6000. Cryo-SEM samples were frozen with a high pressure freezer (Bal-Tec HPM100), freeze-fractured (Bal-Tec BAF060) and SEM was performed on a Zeiss LEO 1530.

## Additional Information

**How to cite this article**: Böni, L. *et al.* Hagfish slime and mucin flow properties and their implications for defense. *Sci. Rep.* **6**, 30371; doi: 10.1038/srep30371 (2016).

## Supplementary Material

Supplementary Video 1

Supplementary Video 2

Supplementary Video 3

Supplementary Video 4

Supplementary Video 5

Supplementary Information

## Figures and Tables

**Figure 1:**
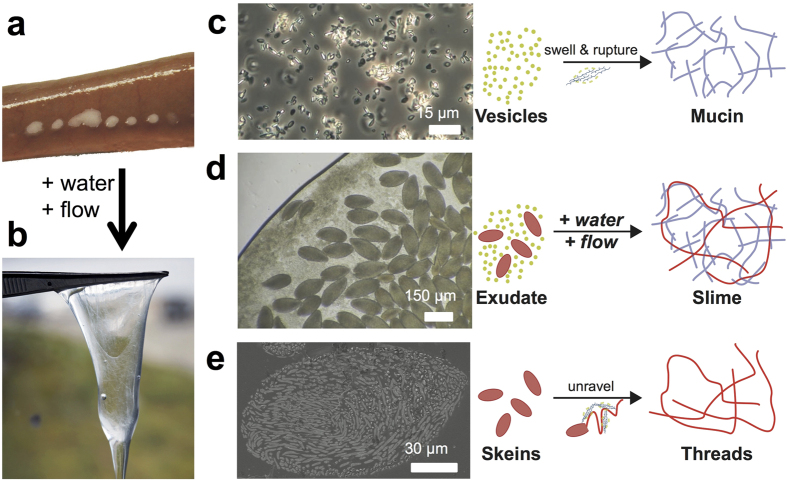
Overview of hagfish exudate components and slime formation in water. (**a**) Exudate is released from pores after electrical stimulation. (**b**) Exudate in water forming hagfish slime. (**c**) Vesicles with mucin stabilized in buffer solution. When in water, the vesicles swell, rupture, and form mucin strands under the influence of flow. (**d**) Microscopy image of hagfish exudate showing both skeins and vesicles with mucin. (**e**) A side view cut Cryo-SEM image of a hagfish skein, which unravels to a single long protein thread when in water. Once both components of the exudate (skeins and vesicles) are released into the seawater, hagfish slime is formed.

**Figure 2:**
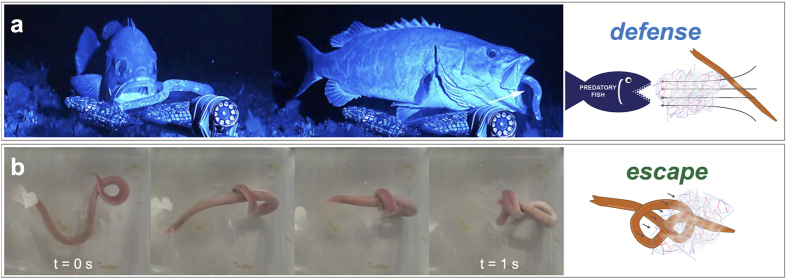
Hagfish defense and escape. (**a**) During predation hagfish instantly form large quantities of slime as a defense mechanism. Predation often occurs through suction feeding where the prey is sucked into the mouth by a strong elongational flow. The arrow depicts jets of slime that were projected into the predator’s mouth upon suction feeding (Adapted and reprinted by permission from Macmillan Publishers Ltd: Scientific Reports, Zintzen *et al.*^14^, copyright 2011). (**b**) Entanglement and self-asphyxiation is avoided by sliding a knot across the body to shear off the slime and escape (see Supplementary Video 1).
