# Supplementary material for: Hagfish slime and mucin flow properties and their implications for defense
Source: Sci Rep. 2016 Jul 27;6:30371. doi: 10.1038/srep30371 (PMC4961968; doi:10.1038/srep30371)
Supplement: Supplementary Information [file srep30371-s6.pdf]

Supplementary information

Hagfish slime and mucin flow properties and their implications for defense

Lukas Böni[1], Peter Fischer[1], Lukas Böcker[1], Simon Kuster[1,*], and Patrick A. Rühs[2]

[1] Department of Health Science and Technology, ETH Zurich, 8092 Zurich, Switzerland
[2] Department of Materials, ETH Zurich, 8093 Zurich, Switzerland

[*]E-mail: simon.kuster@hest.ethz.ch

Supplementary Note:
(a) Calculation of shear rate during knot sliding
(b) List of suction feeding fish preying on hagfish

Supplementary Video 1:
A hagfish is trapped in its own slime. To avoid self-asphyxiation, the hagfish forms a sliding knot to shear off the slime.

Supplementary Video 2:
Complex flow behaviour of natural hagfish slime and sample loading

Supplementary Video 3:
Hagfish mucin viscoelasticity and the effect of shear

Supplementary Video 4:
Extensional elastic properties of mucins in hagfish slime

Supplementary Video 5:
Liquid filament thinning event of natural hagfish mucin in a CaBER (Capillary Breakup Extensional Rheometer), recorded at 5000 fps with a play rate of 24 fps.

Supplementary Note 4:

(a) Calculation of shear rate during knot sliding

Assuming a hagfish body length l = 0.2 m, a knotting time of t = 1 s, and a gap range between the sliding surfaces of h = 0.001 – 0.01 m

This equals a sliding velocity of v = l/t = 0.2 m/s

According to $\gamma = {}^{v}/_{h}$ this corresponds to an average shear rate of $\gamma \approx 20 - 200\ s^{-1}$ during knot sliding.

(b) Selection of suction feeders preying on hagfish

Predator / Predator family	Preys on hagfish	Uses suction feeding
Polyprion americanus (Atlantic wreckfish)	Zintzen et al., (2011)	Brick Peres et al., (2003)
Congridae (Conger eels)	Zintzen et al., (2011)	De Schepper et al., (2007)
Squalidae (Spiny dogfishes)	Zintzen et al., (2011)	Wilga et al., (1998)
Otaria flavescens (Southern sea lion)	Jørgensen et al., (1998)	Berta et al., (2005)
Gadus callarias (Codfish)	Jørgensen et al., (1998)	Muller et al., (1984)
Phoca vitulina (Harbour seal)	Jørgensen et al., (1998)	Marshall et al., (2014)

1. Zintzen, V. *et al.* Hagfish predatory behaviour and slime defence mechanism. *Sci. Rep.* **1,** (2011).

2. Brick Peres, M. & Haimovici, E. Alimentação do cherne-poveiro Polyprion americanus (Polyprionidae, Teleostei) no sul do Brasil. *Atlântica, Rio Gd.* **25,** 201–208 (2003).

3. De Schepper, N., De Kegel, B. & Adriaens, D. Morphological Specializations in Heterocongrinae (Anguilliformes: Congridae) Related to Burrowing and Feeding. *J. Morphol.* **268,** 343–356 (2007).

4. Wilga, C. D. & Motta, P. J. Conservation and variation in the feeding mechanism of the spiny dogfish squalus acanthias. *J. Exp. Biol.* **201,** 1345–1358 (1998).

5. Jørgensen, J. M., Lomholt, J. P., Weber, R. E. & Malte, H. *The Biology of Hagfishes*. (Chapman & Hall, 1998).

6. Berta, A., Sumich, J. L. & Kovacs, K. M. *Marine mammals: evolutionary biology*. (Academic Press, 2005).

7. Muller, M. & Osse, J. W. M. Hydrodynamics of suction feeding in fish. *Trans. Zool. Soc. Lond.* **37,** 51–135 (1984).

8. Marshall, C. D. *et al.* Feeding kinematics, suction, and hydraulic jetting performance of harbor seals (Phoca vitulina). *PLoS One* **9,** 1–11 (2014).
